# Modeling of the hypothalamic-pituitary-adrenal axis-mediated interaction between the serotonin regulation pathway and the stress response using a Boolean approximation: a novel study of depression

**DOI:** 10.1186/1742-4682-10-59

**Published:** 2013-10-05

**Authors:** Oscar Andrés Moreno-Ramos, Maria Claudia Lattig, Andrés Fernando González Barrios

**Affiliations:** 1 Departamento de Ciencias Biologicas, Facultad de Ciencias, Laboratorio de Genética Humana, Universidad de los Andes, Cra. 1a No. 18 A 12 Ed M1, Bogotá, Colombia; 2 Grupo de Diseño de Productos y Procesos (GDPP), Universidad de los Andes, Cra. 1 Este 19 A 40 Ed. Mario Laserna, Bogotá, Colombia

**Keywords:** Serotonin, HPA, Major depressive disorder, Stress, Synchronous boolean networks, BDNF, TRkB

## Abstract

Major depressive disorder (MDD) is a multifactorial disorder known to be influenced by both genetic and environmental factors. MDD presents a heritability of 37%, and a genetic contribution has also been observed in studies of family members of individuals with MDD that imply that the probability of suffering the disorder is approximately three times higher if a first-degree family member is affected. Childhood maltreatment and stressful life events (SLEs) have been established as critical environmental factors that profoundly influence the onset of MDD. The serotonin pathway has been a strong candidate for genetic studies, but it only explains a small proportion of the heritability of the disorder, which implies the involvement of other pathways. The serotonin (5-HT) pathway interacts with the stress response pathway in a manner mediated by the hypothalamic-pituitary-adrenal (HPA) axis. To analyze the interaction between the pathways, we propose the use of a synchronous Boolean network (SBN) approximation. The principal aim of this work was to model the interaction between these pathways, taking into consideration the presence of selective serotonin reuptake inhibitors (SSRIs), in order to observe how the pathways interact and to examine if the system is stable. Additionally, we wanted to study which genes or metabolites have the greatest impact on model stability when knocked out *in silico*. We observed that the biological model generated predicts steady states (attractors) for each of the different runs performed, thereby proving that the system is stable. These attractors changed in shape, especially when anti-depressive drugs were also included in the simulation. This work also predicted that the genes with the greatest impact on model stability were those involved in the neurotrophin pathway, such as CREB, BDNF (which has been associated with major depressive disorder in a variety of studies) and TRkB, followed by genes and metabolites related to 5-HT synthesis.

## Introduction

Major depressive disorder (MDD) is one of the most common debilitating mood disorders worldwide and is becoming increasingly prevalent in children and adolescents. Approximately 15% of the population is estimated to be affected with this disorder at least once in their lifetime [1-4]. MDD has been projected to become the second leading cause of disability worldwide by 2020 according to the World Health Organization (WHO).

MDD is a multifactorial disorder known to be influenced by both genetic and environmental factors. The heritability of MDD is estimated to be 0.37 (95% confidence interval (CI) 0.31-042) based on data from twin studies, and a genetic contribution has also been observed in family studies of individuals with MDD that imply that the probability of suffering the disorder is approximately three times higher if a first-degree family member is affected [5,6].

Childhood maltreatment and stressful life events (SLEs) have been established as critical environmental factors that profoundly influence the onset of MDD [7,8]; however, the question of why some individuals develop MDD when exposed to stressful events and some do not has been difficult to answer. An initial attempt to answer this question was made in 2003 by Caspi et al., who elegantly demonstrated that the risk of developing MDD is higher when an individual has experienced various stressor events during childhood. In this work, they evaluated the possible association of a genetic variant of the serotonin transporter (5-HTT) with maltreatment [9]. This gene x environment interaction approach in which 5-HTT gene variations are proposed to influence the likelihood of stressful life events resulting in MDD has not been fully supported by recent studies, suggesting that other pathways, such as those involved in stress responses, might also be involved in the development of MDD [10,11]. Moreover, one of the most common types of antidepressant used is the Selective Reuptake Inhibitors (SSRIs). SSRIs are designed to block the 5-HTT. Therefore, 5-HT homeostasis in the brain is related to the disease, which makes the 5-HT pathway a strong candidate for studies of MDD [12].

More recently, it has been demonstrated that neurotrophin signaling directly interacts with the serotonin pathway, therefore making it another good candidate for MDD studies. Brain-derived neurotrophic factor (BDNF) is a growth factor involved in regulating the survival and maturation of serotonin (5-HT) neurons during development and in regulating synaptic plasticity throughout life. In the dorsal raphe nuclei (DRN) cell, BDNF activates genes in the serotonin pathway, such as those encoding the serotonin transporter (5-HTT) and the enzymes required for 5-HT synthesis. The 5-HT released from the raphe nucleus cells activate serotonin post-synaptic receptors and autoreceptors such as 5-HT_1A._ Autoreceptor stimulation by 5-HT leads to a blockade of BDNF regulatory genes and, therefore, a decrease in BDNF synthesis [13-15].

The stress response activates a series of processes in the hypothalamus-pituitary-adrenal (HPA) axis culminating in the release of cortisol by the adrenal glands [16]. The glucocorticoid receptor is expressed in serotoninergic neurons [17-19], which is indicative of glucocorticoid-exerted control in these neurons. On the other hand, both stress and glucocorticoids are known to decrease brain levels of BDNF in rodents and post-mortem human tissue [20]. Taken together, this information suggests the existence of a cycle involving these three pathways (the 5-HT regulation pathway, the stress response pathway and the BDNF regulation pathway), which are essential to enable the serotonin system to respond to various stimuli throughout life.

We propose a Boolean approximation to analyze the HPA axis-mediated (stress response) interactions between the 5-HT regulation pathway and the neurotrophin signaling pathway. Synchronous Boolean networks (SBNs) have proven to be a valuable model for understanding how a large number of components (nodes) in a network interact with each other. The logic of SBNs is based on the use of binary values, 0 as “inactive” and 1 as “active”, to model a complex system [21,22]. SBNs are networks that update their nodes synchronously in a discrete time step [23]. Since we are analyzing a large complex network (41 nodes), we chose synchronous update since this type of modeling changes expression, activation or inactivation of different nodes simultaneously in a discrete time [24], feature observed in various biological systems [25,26]. Moreover, as we are dealing with a large network, this updating approximation is computationally feasible.

Through this type of network modeling, it is possible to understand and annotate complex pathways. SBN approximations have been used to model the orientation pathways by which neural crest stem cells give rise to two different types of sensorial cells [27], and it has also been used to model biofilm formation in the *E. coli* strain K-12 [28]. Furthermore, this approach has also been applied to search for new candidate genes in schizophrenia [21] and as a modeling technique in cancer studies [29].

The aim of this work was to use a Boolean approximation to analyze an integrated network involving the 5-HT neurotransmitter pathway, neurotrophin signaling and the HPA cortisol synthesis pathway in the presence and absence of stress and serotonin selective reuptake inhibitors (SSRIs). We also evaluated network stability and the effects that knocked-out genes had on the network to search for probable candidate genes involved in MDD.

## Methods

**Figure 1:**
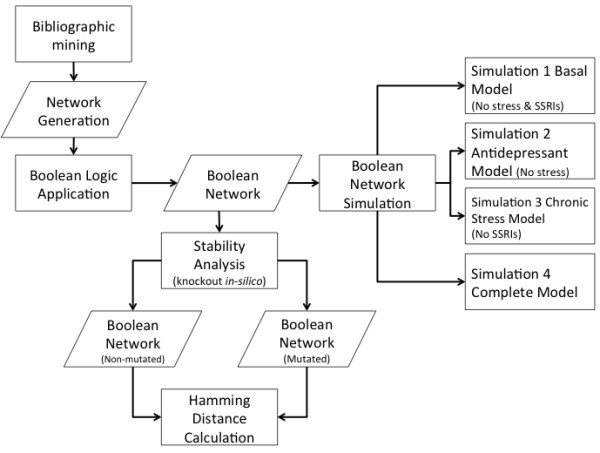
**A flow chart illustrating the methodology used to model the network.** For more information, refer to the Methods section.

The Methods section is depicted in Figure 1 to clarify the methodology used.

### Model definition and network simulation

The biological information used to generate the network is shown in Appendix A and was analyzed using an SBN approximation. The model was simulated using the Random Boolean Networks (RBN) toolbox (free download at http://www.teuscher.ch/rbntoolbox) for Matlab® by using the tools that allow for well-defined connections among nodes. Boolean logic was applied to identify the logic operators (“AND” and “AND-NOT”) that allow the model to simulate the network [30]. The Boolean simplification gave 41 nodes that were logically connected and allowed the construction of a rules-matrix, which defines the logic transition rules for each node in the network, and a connection-matrix, which explains the connectivity of the nodes. Both matrixes are in conjunction the mathematical model behind the simulations performed.

The rules-matrix size was 2^k^xN (N nodes and k connections). Each node has k possible entrances that only generate two responses (1 or 0 for on or off, respectively). Our network has 41 nodes and up to 4 entrances with a rules-matrix size of 2^4^x41. Each column of this matrix is created using 41 different matrices, where each of these matrices holds the response of each node according to the 4 different binary organized entrances. The connection-matrix created has a size of NxN where each of the matrix entrances (i,j) defines the number of connections from node i to node j with a column sum restriction equal to k.

The initial states for all nodes were set to 1 (on) for every node in the network except for the nodes corresponding to stress and to SSRI, which were permuted between 1 and 0 (on or off). Therefore, four initial states were generated: 1) Basal Model: all 41 nodes initially active except the stress and SSRI nodes, 2) Antidepressant Model: all 41 nodes active except the stress node, 3) Chronic Stress Model: all 41 nodes active except the SSRI node and 4) Complete Model: all 41 nodes active. In our model, the stress, tryptophan (TRP) and selective serotonin reuptake inhibitor (SSRI) nodes remain in a steady state throughout the simulations because they are not downregulated by any other node. To verify that the network was stable, attractors were obtained from each simulation. The simulations performed are shown in Figure 1. Each of the four simulations were performed in a 2.8GHz Intel Core 2 Duo with 4GB RAM, taking ~5 s per run.

### Stability analysis through *in silico* knockouts

*In silico* knockouts were generated for all nodes and their effects on network stability (Convergence/divergence from the same initial condition in a discrete time [21]) were evaluated by comparing two networks (mutated and non-mutated) simulated in parallel. In the two networks, the state of SSRI was fixed to 0 (i.e., switched off) because it is not a normal biological component of the pathway. Therefore, the possible states for the two networks were reduced from 2^41^ to 2^41-1^. The simulations performed were run with the same discrete time span (t = 100) to allow calculation of the normalized Hamming distance (number of positions, in a vector or matrix, at which the corresponding component is different divided by the number of total components) between each of the states at the same discrete time throughout the whole time span (100 hamming distances calculated). Even though several metrics are used to infer difference/similarity between two objects, we consider normalized Hamming distance as an adequate measure to observe the convergence/divergence, at the same discrete time, between the two models (mutated and non-mutated) [21].

The initial states were randomly selected but they were the same for both networks, which allowed a statistical measurement of convergence/divergence of the network dynamics [21]. Both networks were run 1000 times, and the mean normalized Hamming distance and its standard deviation were calculated and plotted against the discrete time to determine the perturbation when the genes in question were knocked-out. Even though we know that there are other metrics to infer difference/similarity between two objects, we consider normalized Hamming distance as an adequate measure to observe the convergence/divergence, at the same discrete time, between the two models (mutated and non-mutated) [21]. The biological reason behind this approximation is that most genetic diseases are a product of a cascade block that leads to an undesired stable state.

To evaluate the accuracy of the mean normalized Hamming distances, a bootstrapping method was used to calculate the mean deviation error and thereby validate the statistical significance as the data did not fit a defined probability distribution [31,32]. All the simulations were performed in a single code line in a 2.8GHz Intel Core 2 Duo with 4GB RAM, taking ~7.4 h.

## Results and discussion

### Model definition: genetic pathway and Boolean logic applied to the model

**Figure 2:**
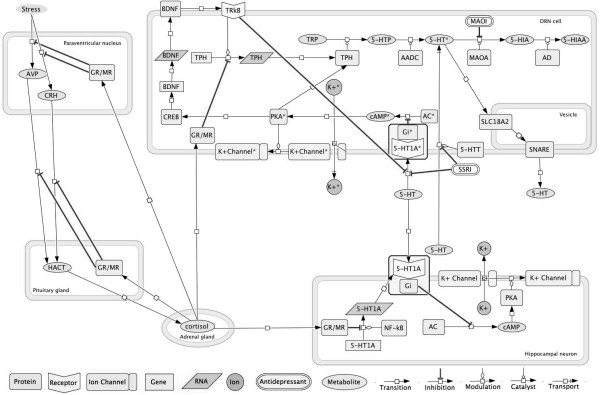
**The genetic pathway predicted to regulate the interaction between 5-HT synthesis, transport and degradation and the stress response mediated by the HPA axis.** This figure was generated using the computational program CellDesigner 4.1. * Nodes located in the dorsal Raphe nucleus (DRN) cells.

**Figure 3:**
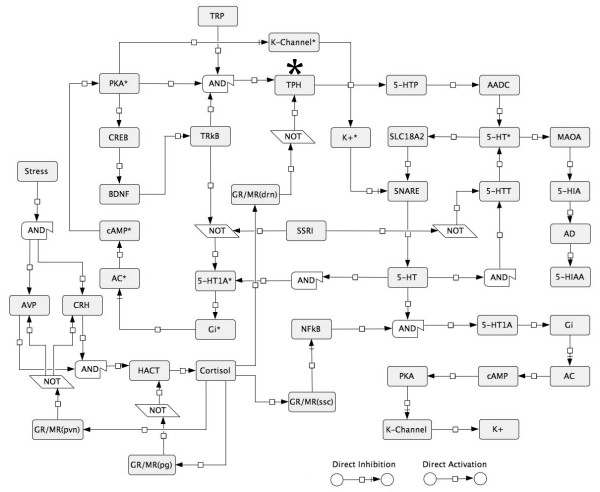
**A simplified model of the regulatory interactions between the serotonin and stress response pathways mediated by the HPA axis.** AND/NOT operators are included to show the Boolean logic used. When no operator is shown between the nodes, the activation state is given by any of the nodes upstream. * Nodes located in the dorsal Raphe nucleus (DRN) cells.

The network was constructed using the DRN cell and hippocampal serotonin-sensitive cell brain areas in combination with the stress response pathway involving the periventricular nucleus, the pituitary gland and the cortisol-secreting adrenal gland (Figure 2). The network was simplified using a Boolean approximation as depicted in Figure 3.

### Network simulation

**Figure 4:**
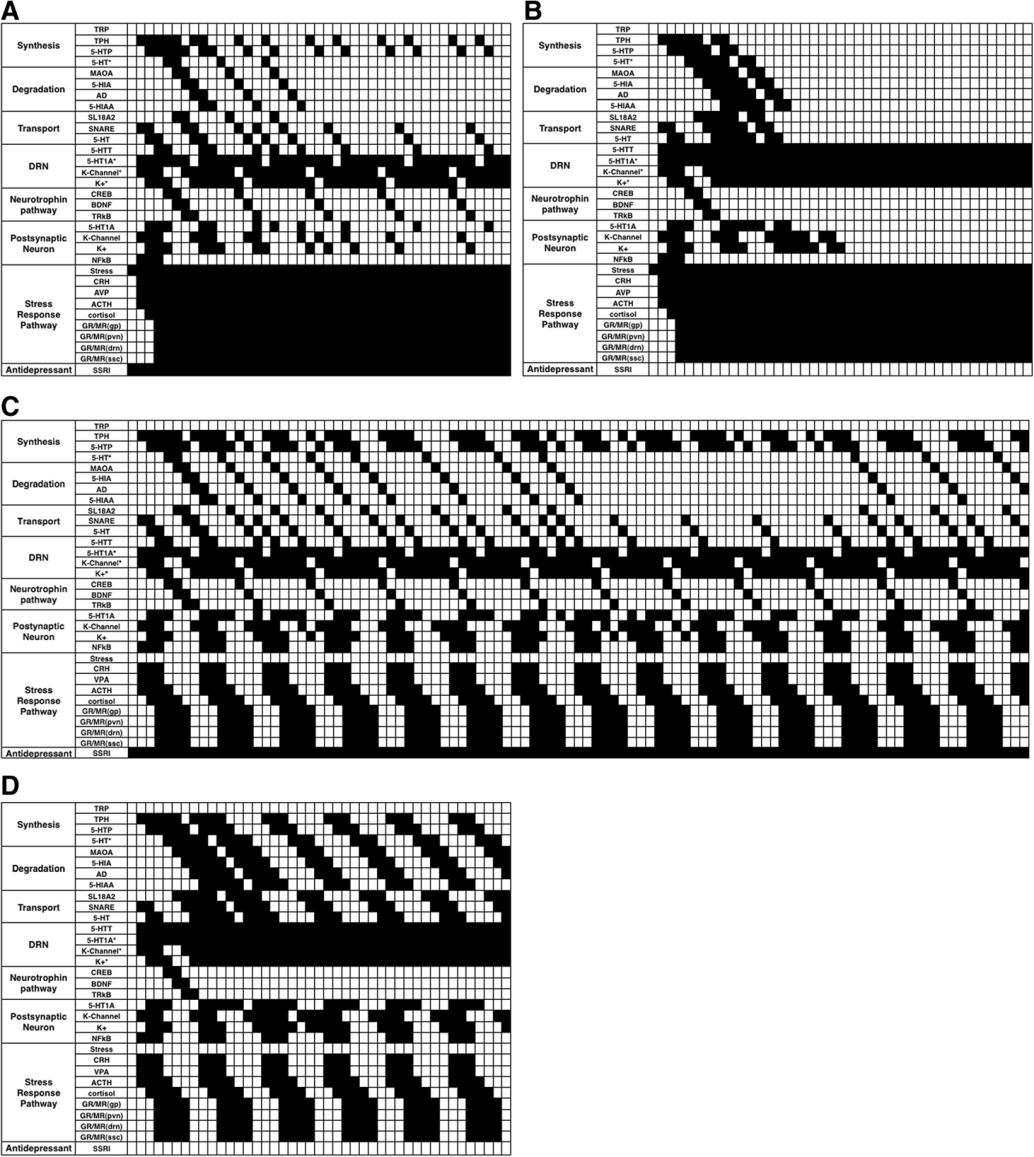
**Results obtained for all the runs performed with the model generated by a Boolean approximation using Synchronous Boolean Networks (SBNs).** Nodes related to G-protein signaling are not shown. An input vector of ones was used for all nodes except the SSRI and stress nodes, which were activated or deactivated to generate 4 possible simulations. **A**. The pattern obtained when stress and SSRI were both in the ‘off’ state (basal system) and the other nodes were ‘on’. **B**. The effect of SSRI activity on the serotonin regulation pathway under unstressed conditions is shown. Notice that the antidepressant activity blocks 5-HTT and 5-HT_1A_^*^, causing 5-HT synthesis, degradation and transport pathways to remain active. **C**. The pattern generated when the stress node is ‘on’ and SSRI is ‘off’. In this case, a cyclical pattern was observed for nodes related to 5-HT synthesis, transportation and degradation. This figure also shows that while the stress response is well regulated, a persistent stressed state activates the HPA axis over time. **D**. SSRI clearly regulates the cyclical pattern shown in **C**, but it does not cause the same pattern shown in **B**. This is in line with expectation and demonstrates that SSRIs do not have any effect when the patient is stressed.

The four simulations (Figure 1) demonstrated network stability by means of an attractor (cycling pattern). Figure 4 depicts the results of the four simulations described in Figure 1 and Methods section. It indicates that all four models show attractor stability. Each of the individual models was described separately below (Section 2, subsection 2.2.). The nodes involved in G-protein signaling (in both presynaptic and postsynaptic neuron) are not shown since they are ubiquitously expressed. Thus, no effect is observed when deleting these nodes. This reduces the nodes shown in Figure 4 from 41 to 32. Hence, G-protein signaling will not be a cause of MDD.

In the Basal Model (Figures 1 and 4A), we found that the approximation is reliable because the autoreceptor (5-HT_1A_^*^) turns 5-HT synthesis and transportation pathways off, and this finding agrees with those of previous *in vivo* studies [33-35] (see *Appendix A* for pathway details).

The Antidepressant Model (Figure 1) demonstrates active synthesis, transport, degradation and activation of the hippocampal serotonin-sensitive neuron (Figure 4B). A limitation to Boolean modeling is that it is concentration independent and therefore it cannot take into account the complex kinetics involved in controlling the serotonin system in vivo. It is believed that SSRIs block the serotonin transporter and thereby increase 5-HT concentrations in the synaptic cleft, which in turn leads to a gradual desensitization of the 5-HT_1A_^*^ autoreceptor [33-36]. This interaction between synaptic 5-HT and the 5-HT_1A_^*^ autoreceptor is impossible to observe in a Boolean simulation because, as stated above, no data on concentrations or kinetics can be used.

Interestingly, in this simulation we did observe a constant synthesis of 5-HT when administering SSRIs (Figure 4B). This result suggests that other pathways might be interacting with 5-HT, as has also been implied by the results of other studies [37,38]. However, care should be taken when considering this possibility because the model cannot consider the effects of 5-HT concentration on its homeostasis.

The Chronic Stress Model (Figure 1) considers a stressor event in the absence of SSRIs (Figure 4C). It is well known that stress events activate the HPA axis (Figure 2). The nodes related to the stress response mediated by the HPA axis (VPA, CRH, HACT, GR/MR_(pg)_ and GR/MR_(pvn)_), indicate a strong regulation that is evident in the constant cycling pattern that exists while the stress node is in the ‘on’ state. Furthermore, this stress response model shows that the cortisol receptor in serotonin DRN cells blocks TPH and deregulates the serotonin cyclic synthesis and transport pathways. This strong negative effect was observed in the model 57 discrete times as a longer attractor and has been reported to affect serotonin by blocking its synthesis through the activity of increased levels of cortisol [39]. Finally, the Complete Model (Figure 1) considered both the SSRI effect and the stress response (Figure 4D). We observed that the effect of SSRIs does not produce the same long cyclic pattern that was generated when stress alone was present (Figure 4C). The pattern is more repetitive than that observed when stress was active, but it has no similarities to the Basal Model simulation (Figure 4A). The pattern shows that SSRIs regulate the serotonin pathway when chronic stress is present, but it does not allow for the constant presence of 5-HT in the synaptic cleft. Accordingly, here we show that SSRIs act differently depending on the presence or absence of chronic stress, indicating that stress deregulates the effect of the SSRIs.

Even though the present work did not consider the effect of stress on neural plasticity and connectivity, it is well known that treatment with SSRI-type antidepressants seems to modulate hippocampal neurogenesis, which is essential in MDD treatment [40,41]. Hence, this work leaves the door open to future simulations to consider not only the 5-HT regulation and BDNF regulation pathways but also the BDNF mechanisms that modulate neural plasticity in serotoninergic neurons.

### Stability analysis through knockouts *in silico*

**Figure 5:**
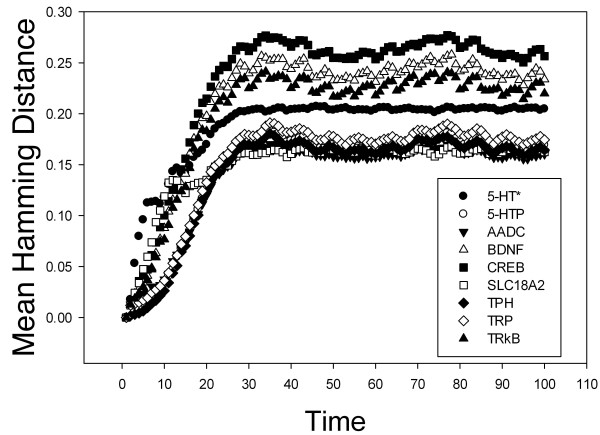
**The effects of knocking out key nodes in the model.** The importance of individual nodes was determined based on the mean Hamming distance calculated from 100 discrete runs following node knockout in the model. The nodes corresponding to CREB, BDNF and TRkB had the greatest negative effect on network stability when knocked out, while those related to 5-HT synthesis had the next greatest negative effect.

**Figure 6:**
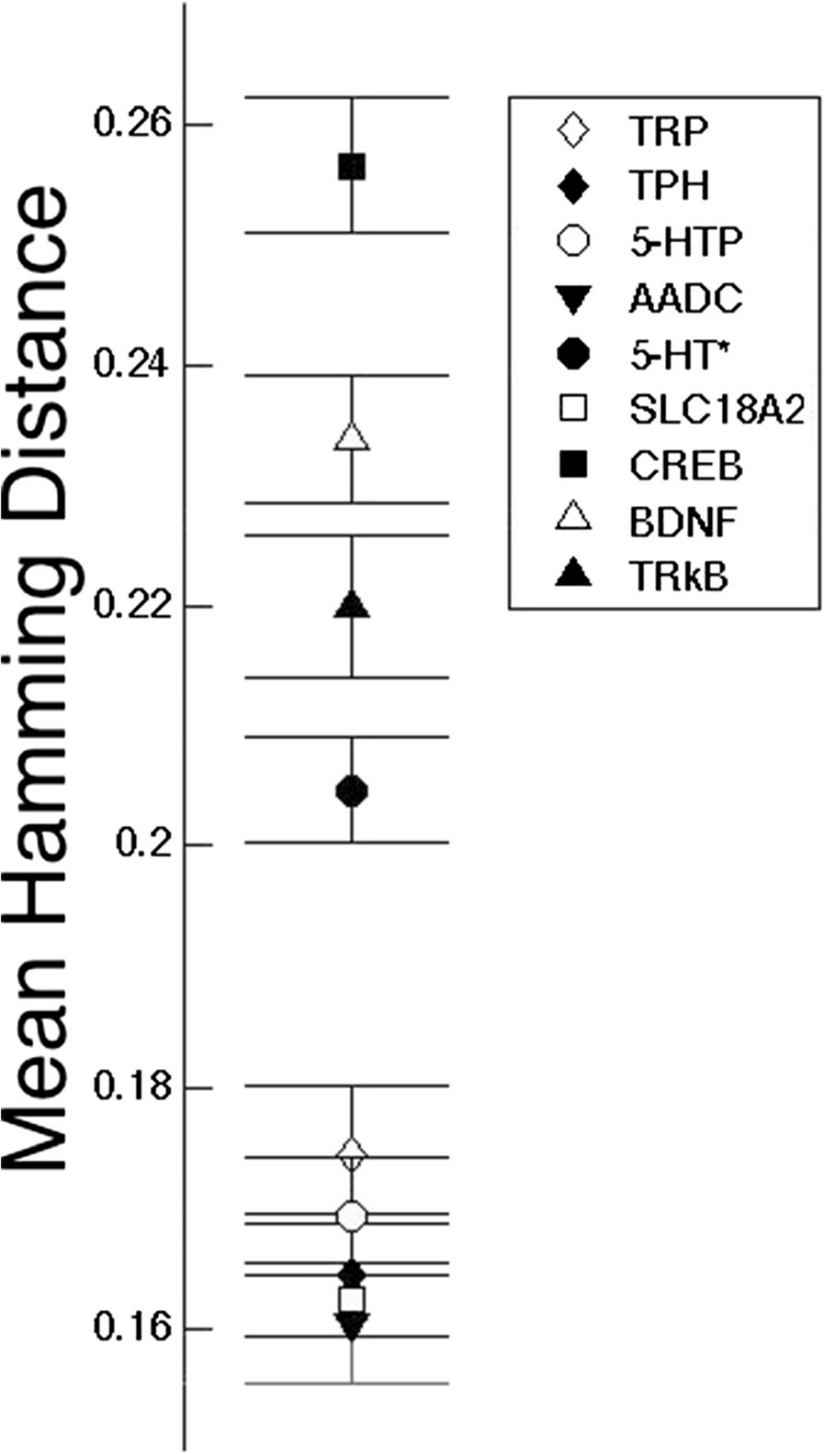
**Results obtained by the application of the bootstrapping method to calculate the deviation error (only shown for the 100th time step because the system has reached stability).** These results show strong similarity to those obtained by calculation of the mean Hamming distance (Figure 5).

In our network stability analysis, 27 nodes where initially considered (Table 1). However, because 4 of these nodes corresponded to glucocorticoid receptors, they were treated as a single node in our analysis. Therefore, 23 nodes were evaluated independently for their effects on network stability. Knock-out networks were simulated in parallel with wild type networks. When the Hamming distance and its mean were plotted versus discrete time, the results indicated that the nodes corresponding to TrkB, BDNF and CREB have the greatest effects on network stability (Figure 5). Although the data indicate that the BDNF and 5-HT synthesis pathways have an impact on network stability, it is important to consider that they are the mean Hamming distances reported when those nodes were *knocked-out*. Consequently, we decided to evaluate the accuracy of the results by calculating the deviation error using the bootstrapping method. Figure 6 confirms that nodes related to the BDNF pathway and those related to 5-HT synthesis generate a disorder in the network when they are *knocked out*, thereby reinforcing the theory that genes related to the BDNF pathway (CREB, BDNF and possibly TRkB) are high-impact risk factors involved in the development of MDD.

**Table 1:** Genes selected for knockout analysis

**Node Name**	**Gene or Metabolite**
TRP	Tryptophan
TPH 2	Tryptophan hydroxylase 2
5-HTP	5-hydroxytryptophan
AADC	Aromatic L-amino decarboxylase
5-HT*	Serotonin in the DRN
MAOA	Monoamine-oxidase A
SL18A2	Solute carrier 18A-2
5-HT	Synaptic serotonin
5-HT1A	Postsynaptic 5-HT receptor 1A^a^
K-Channel	Presynaptic potassium channel ^a^
K+	Postsynaptic potassium
5-HT1A*	Presynaptic 5-HT receptor 1A^a^
K-Channel*	Presynaptic potassium channel ^a^
K + *	Presynaptic potassium
5-HTT	Serotonin transporter
CREB	cAMP-response element binding
BDNF	Brain-derived neurotrophic factor
TRkB	Tyrosine kinase B
CRH	Corticotropin releasing hormone
VPA	Vasopressin
ACTH	Adrenocorticotropic hormone
cortisol	Cortisol
GR/MR_(PG)_	Glucocorticoid/Mineralocorticoid receptor (pituitary gland)^b^
GR/MR_(PVN)_	Glucocorticoid/Mineralocorticoid receptor (paraventricular nucleus)^b^
GR/MR_(DRN)_	Glucocorticoid/Mineralocorticoid receptor (dorsal raphe nucleus)^b^
GR/MR_(SSC)_	Glucocorticoid/Mineralocorticoid receptor (sensitive serotonin cell)^b^
NFkB	Nuclear factor

^a.^ Gene knocked out in a specific tissue, ^b.^ gene knocked out globally, * presynaptic node expressed in the dorsal raphe nucleus. (DRN) dorsal raphe nucleus, (PG) pituitary gland, (PVN) paraventricular nucleus, (SSC) sensitive serotonin cell.

Various authors have reported low BDNF serum concentrations in depressive patients and in association with suicidal behavior [42-44]. BDNF administration to the DRN cells has been observed to have an antidepressant effect [45]. Additionally, the Val66M Met polymorphism in BDNF has been associated with depressive states [46]. Figures 5 and 6 also show that if any of the nodes related to either the 5-HT synthesis pathway or transport to the synaptic cleft is mutated, there is an effect on system stability. This may explain why patients with MDD synthesize lower levels of serotonin than healthy controls [47].

In our model, we considered the neural connections that are already formed in an adult. Thus, the BDNF biological functions examined are not related to neural plasticity but instead relate to the DRN serotoninergic phenotype. In the model, stress does not downregulate BDNF expression in DRN cells (Figures 4A and 3C). However, an interaction between stress and the expression and biological functions of BDNF, mostly at early developmental stages, has been described [48]. In fact, it has been found that the cross-sectional area of the DRN is decreased in MDD patients, possibly due to a neuropil reduction [49]. Neuropil reduction is related to low BDNF and TRkB concentrations [50], and stress may therefore affect BDNF regulation pathways in the DRN cells. Further studies are needed to confirm this possibility.

A very important finding is the identification of the TRkB receptor as another potential risk factor for MDD. TRkB mRNA has been found to be expressed in the DRN, as has its ligand, BDNF, and both are essential to the serotoninergic phenotype of the cells [49,51]. Recently, a variety of SNPs in TRkB have been found to be associated with MDD or with attempted suicide in German and African American populations [52]. Moreover, glucocorticoids reduce TRkB protein concentration in the prefrontal cortex and the hippocampus in the mouse brain, which results in the development of depressive-like behavior and anxiety [53]. Therefore, we consider this a plausible candidate for future MDD association studies in different populations or other simulation studies where other tissues and pathways are taken into account.

## Conclusions

We constructed a stable model of depression. In this model, stress modifies the serotonin regulation pathway as predicted. The effect of antidepressants observed in the model agrees with expectations because the model incorporating SSRI activity exhibited increased DBNF activity even when stress was also an included factor; however, SSRI activity cannot normalize the regulation pattern when the patient has an acute stress event.

The patterns shown have been derived from biological data and are an excellent tool for visualizing the complex system that they model. However, the system is unable to accurately model the pathway in its entirety due to the lack of sufficient biological information. Thus, we propose that the BDNF regulatory pathway, especially TRkB, must be studied in more detail to clarify some aspects of MDD etiology.

## Appendix A

The genetic regulation network obtained was initially based on the interaction pathway found in http://www.pantherdb.org with the accession number P04375 [54]. We performed a thorough bibliographic mining following the procedure described in [55]. The network generated is described briefly below.

The serotonin neurotransmitter (5-HT) is synthesized in the DRN cells by tryptophan (TRP) hydrolysis mediated by the enzyme tryptophan hydroxylase (TPH). This reaction produces 5-hydroxytryptophan (5-HTP), thus becoming the main source of 5-HT. Serotonin can be degraded by monoamine oxidase A (MAOA) to produce 5-hydroxyindoleacetic acid (5-HIAA) [56-60]. Serotonin vesicular packaging is mediated by the solute carrier 18A-2 (SLC18A2), and its liberation into the synaptic cleft is performed via the SNARE complex [61,62].

Synaptic 5-HT can activate the 5-HT receptors (especially the postsynaptic receptor 5-HT_1A_) of the 5-HT-sensitive hippocampal neurons. This is followed by activation mediated by G_i_ proteins and leads to cell hyperpolarization [33,63-65]. High synaptic 5-HT concentrations lead to DRN 5-HT_1A_ autoreceptors activation which in turn lead to DRN neuron hyperpolarization. This hyperpolarization causes a reduction in 5-HT synthesis that results from a blockade of TPH transcription by BDNF pathway activity and a reduction in the transport of 5-HT [66-68].

The serotonin transporter (5-HTT), which is expressed by DRN cells, recaptures 5-HT from the synaptic cleft back into the presynaptic neuron, thereby lowering the synaptic neurotransmitter concentration when it is excessive. This transporter is one of the main targets for SSRI antidepressants, which act by blocking 5-HT recapture and lead to higher 5-HT synaptic concentrations [34,35,68]. When the synaptic 5-HT concentration is higher than normal, the autoreceptor 5-HT_1A_ is desensitized, and this leads to constant synthesis and transport of 5-HT in DRN cells [36,69].

The synthesis and downregulation of 5-HT in the DRN is also controlled by the brain derived neurotrophic factor (BDNF). Normally, TRkB is activated when BDNF is expressed, thereby blocking 5-HT_1A_ autoreceptor synthesis and increasing TPH transcription, which leads to an increase in activation of cAMP and PKA. When PKA is active, it promotes BDNF transcription via phosphorylated CREB [13-15].

Regarding the stress response, stress activates the HPA axis starting with corticotropin releasing hormone (CRH) and vasopressin (AVP) in the periventricular nucleus (PVN), which is located in the hypothalamus. Both hormones are transported by the blood stream to the pituitary gland (PG) where they activate the synthesis of adrenocorticotropic hormone (ACTH). ACTH is then transported by the blood stream to the suprarenal cortex where it mediates cortisol synthesis [16,41]. When cortisol levels rise in the blood stream, they cause negative feedback regulation of cortisol synthesis through activation of glucocorticoid receptors (GRs) and mineralocorticoid receptors (MRs) [41]. Cortisol has a negative effect on the DRN and the hippocampal 5-HT-sensitive cells. In the DRN, cortisol blocks TPH transcription by activating GRs and MRs [39]. Cortisol also activates GRs and MRs in the hippocampus, and this blocks the function of the transcription factor NF-κB, which in turn leads to weak transcription of the gene encoding the 5-HT_1A_ receptor [70].

## Competing interests

The authors declare that they have no competing interests.

## Authors’ contributions

OAM provided the study idea, performed the modeling and simulations, and wrote the manuscript. MCL revised the biological basis of the model generated and revised the manuscript. AFG revised and checked the modeling approximation, and revised the manuscript. All authors read and approved the final manuscript.
